# Nutritional implications of alternative proteins: a commentary

**DOI:** 10.1017/S1368980025000242

**Published:** 2025-03-17

**Authors:** Ursula Davis, Jack Bobo, Paul Wilson, Peter Noy, Roberto Mansilla, Gavin Long, Simon Welham, John Harvey, Evgeniya Lukinova, Georgiana Nica-Avram, Gavin Smith, David Salt, Andrew Smith, James Goulding

**Affiliations:** 1 Centre for Food Policy and Foresight, Food Systems Institute, University of Nottingham, Nottingham, UK; 2 N/LAB, Nottingham University Business School, Jubilee Campus, University of Nottingham, Nottingham, UK; 3 Division of Food, Nutrition and Dietetics, School of Biosciences, Sutton Bonington, University of Nottingham, Nottingham, UK; 4 Future Food Beacon of Excellence and School of Biosciences, Sutton Bonington, University of Nottingham, Nottingham, UK

**Keywords:** Alternative proteins, Dietary transition, Nutritional implication, Dietary implications

## Global dietary transitions

Global food demand is expected to increase by 50 % to 60 % between 2019 and 2050^(1,2)^. Meeting this demand through expanded production of animal protein would come at a high cost for the environment. Alternative protein products may present an alternative to meet this demand. The global protein transition, i.e. the shift from animal protein to alternative protein sources to meet human nutritional needs, prompts a complex debate around food availability and the cultural, health and environmental implications of such a transition. This commentary focuses on illustrating the nutritional effects of global dietary change through alternative protein consumption, rather than debating the overall global protein transition.

Demand for alternative proteins including plant-based proteins such as soya and pea, mycoproteins and proteins produced through precision fermentation has been on the rise. This rise is driven by mounting concerns surrounding the environmental impact of animal-derived proteins^(3)^, diet-driven health considerations^(4,5)^ and ethical concerns surrounding animal welfare within the traditional food system. This transition is reflected in consumer behaviour and ultimately the recent growth of the alternative protein market. While early projections suggested that by 2035 alternative proteins could account for 11 % of the total protein market^(6)^, growth slowed dramatically in 2023, putting in doubt such optimistic projections. Nevertheless, the need for an alternative to expanded livestock production remains, and the potential for renewal exists, underpinned by increased investment in food technology, leading to more diverse and palatable options in the plant-based protein sector^(7)^.

Global protein transitions can be driven by several factors. Dramatic increases in livestock production to meet future protein demand would come with a further and significant increase in livestock’s environmental footprint^(8)^. As a result, consumers may adapt their diets in response to environmental considerations^(9,10)^. Within this school of thought, alternative proteins may offer a more sustainable alternative. Health considerations also play a pivotal role. Some consumers associate plant-based diets with wellness and disease prevention^(5)^. Additionally, ethical concerns regarding animal welfare have led to growing demand for animal-free food options. Together, these trends may result in a paradigm shift in food consumption, with potential long-lasting impacts on the environment, nutrition and food policy.

Many food debates discuss the nutritional implications of dietary changes, including current discussions on the role of ultra-processed food in the obesity epidemic^(11,12)^. Yet, little research to date has focussed on the potential nutritional implications of wide-scale alternative protein adoption^(13)^. As alternative protein consumption increases, public nutrition and health policy must also adapt. Public policy must be underpinned by rigorous and robust scientific research with respect to the nutritional implications of the replacement of animal proteins with alternative proteins.

## Nutritional adequacy of diets

Many consumers report an interest in decreasing consumption of animal products and increasing consumption of plant-based products^1^. Environmentalists highlight the sustainability benefits of plant-based and alternative protein diets as potentially having a reduced environmental impact in comparison to their animal protein counterparts^(14)^. Indeed, the IPCC recommendations highlight the role of reducing meat consumption and food waste in mitigating climate change^(15)^. Arguments for plant-based and alternative proteins hinge on the idea that the high environmental cost of producing animal-based foods justifies the transition to plant-based alternatives.

However, consumers may not be aware of the nutritional differences between animal products and their substitutes, some dieticians and nutritionists have identified a risk of deficiencies in the adoption of such diets. Concerns arise around potential deficiencies in vitamin B_12_^(16)^, Fe^(17)^, *n*-3 fatty acids^(18)^ and Ca^(19)^, all of which are abundant to varying degrees in animal proteins. These concerns are particularly pronounced for specific groups such as pregnant women, children and the elderly, who have distinct nutritional needs.

Health considerations must play a pivotal role in a nutritious dietary transition. In many cases, the food industry has been responsive to these challenges as demonstrated through increasing investment in fortifying plant-based products with essential nutrients^(20)^. This ultimately reflects a growing recognition of the need to balance the environmental sustainability of food choices with nutritional adequacy.

## Research driving public policy

Rigorous scientific research is necessary to guide policy decision-making that ultimately benefits society. Research, including the EAT-Lancet report, has explored the potential of plant-based diets to lower emissions. While studies have raised concerns about the ability of some communities to access or afford diets recommended in the EAT-Lancet report, less attention has been paid to the nutritional implications of large-scale dietary transitions to alternative proteins.

The work of Mansilla *et al.* ^(13)^ is an exemplar of how alternative protein transition research can be leveraged to inform public policy. The authors study the nutritional changes associated with shifting from dairy to plant-based milk alternatives using large-scale shopping data (*n* 512 397 frequent customers) from Co-op Food, UK. This swap is one that may carry notable nutritional implications, particularly in the case of iodine. Iodine is an essential nutrient, primarily obtained from dairy and seafood, and is required for the production of thyroid hormones which regulate metabolism and numerous health-critical physiological functions. In the UK, milk is the principal source of iodine,^(21)^ and currently, many alternative milks are not fortified with iodine. As the replacement of dairy milk with alternative milks increases, there exists a risk that many individuals will experience iodine deficiencies. The findings of the study showed that 83 % of consumers switching from dairy to plant-based milk alternatives experienced a substantial decrease in weekly iodine purchasing, with 57 % of consumers showing more than a 50 % reduction. Whilst the study does not aim to provide a demographic make-up of the cohort, it highlights the potential extent of iodine inadequacy amongst consumers of plant-based alternatives.

The scale of this study is such that it supports traditional methods of dietary tracking and provides robust findings that can be utilised in the development of public health strategies. In this case, the study shows the potential benefit of developing a public health strategy focusing on the guidelines for fortification of plant-based dairy replacements.

### Broader implications

This study raises questions about the nutritional implications of the broader protein transition with respect to meat analogues. Meat analogues are intended to replace or reduce the consumption of traditional meat products but may not deliver the same nutrient profile as meat from animal products which provide a complete source of protein. As a result, the growth of meat analogue products could have nutritional implications. Manufacturers of such products must exercise care in marketing their products by including transparent nutrient product profiles and highlighting nutrients that could be missing compared to traditional counterparts.

This transition away from animal-derived products to plant-based alternatives has implications across sections of society. Indeed, the implications for children, pregnant women and the elderly are of great importance. These groups are amongst the most vulnerable across society, and each has particular dietary needs. The implications of dietary transitions must be carefully understood. For children, the shift away from animal products can significantly impact their intake of essential nutrients. Developmental delays in growth, bone formation and cognitive function from inadequate consumption of key nutrients such as Fe^(22)^, Ca, vitamin D and iodine can have lifelong impacts. The risk that plant-based diets might not provide these nutrients in sufficient quantities necessitates careful meal planning and/or supplementation.

Similarly, pregnant women relying on plant-based diets must be careful to ensure they receive sufficient nutrients like iodine, Fe and Ca as they are essential for foetal development. Severe cases of iodine deficiency, for instance, can lead to permanent, unrecoverable outcomes such as cretinism in newborns^(23)^, while insufficient Fe intake can cause anaemia, increasing the risk of spontaneous abortion, foetal death preterm delivery and low birth weight^(24)^.

The elderly face challenges with nutrient absorption, and deficiencies in Ca and vitamin B_12_ are common concerns^(25,26)^. Vitamin B_12_ deficiency is of concern as it can lead to anaemia and neurological issues. Ca is critical to maintaining bone health and its deficiency can lead to osteoporosis. Given that these nutrients are typically abundant in animal products, consumers eating plant-based diets might require fortified foods or supplements. In addition, demographic projections show that over 25 % of the UK population will be over 65 years old within the next 50 years, making the UK particularly susceptible to health consequences from the dietary change described here^(27)^.

Currently, a third of over 65-year-olds experience bone fractures annually (over 300 000 people), and the prevalence of osteoporosis is increasing and disproportionately impacts females. In addition to the implications on Ca intake, a reduction in animal protein consumption/replacement by alternatives such as plant-based milks and meat analogues may result in reduced protein intake, further exacerbating the risks of bone fractures. Further research is needed to better understand the potential impacts of such dietary changes, including the nutritional implications of low- or no-meat diets. This evidence could inform biofortification or supplementation approaches for at-risk demographics.

In summary, while plant-based diets might offer health benefits in some situations, they also require careful consideration to meet vulnerable populations’ dietary needs. This includes strategies like fortification, supplementation and tailored dietary planning.

## Policy recommendations

Policy interventions play a crucial role in addressing the potential nutritional challenges associated with the widespread adoption of plant-based alternative proteins. This section outlines policy recommendations to mitigate the potential risks associated with dietary shifts, focusing on nutritional labelling, food fortification, dietary guidelines and the role of healthcare providers. These recommendations are intended to support a balanced approach to the possible growth of plant-based diets and alternative proteins, particularly considering the needs of vulnerable populations.

### Nutritional labelling and consumer education

Enhancing nutritional labelling on alternative protein products is critical. Labels should indicate nutritional content, including the presence and levels of essential nutrients. Public health campaigns are equally important and should focus on educating consumers about the nutritional aspects of plant-based diets, emphasising the importance of a balanced diet. These campaigns can utilise various media channels to reach a broad audience and should be tailored to the specific needs of different population segments.

### Food fortification and dietary guidelines

Developing policies for fortifying plant-based products is a key strategy. This might include requiring the addition of nutrients like iodine, Ca and vitamin B_12_ to plant-based foods that naturally lack these essential elements. These guidelines should ensure nutritional equivalence between replacement products and their traditional counterparts which are nutritionally rich and bound by legislation. Updating dietary guidelines to reflect the nutritional profile and needs associated with plant-based diets is also crucial. Guidelines should offer clear, practical advice for maintaining balanced nutrition while adhering to a plant-based diet with regular revision to reflect the latest scientific findings.

### Role of healthcare providers

Healthcare providers play a pivotal role in navigating dietary transitions to plant-based diets. They need to be well-informed about these diets’ nutritional benefits and potential deficiencies. This knowledge is essential for providing accurate dietary advice, especially to vulnerable groups as detailed previously. Training and resources for healthcare providers should be updated regularly to keep pace with the latest research and dietary trends. This will enable them to offer evidence-based recommendations on diet and supplementation where necessary.

### Conclusion

As we consider the possibility of a global transition to more alternative proteins within global protein consumption, there is an urgent need to concurrently develop public policy to guide this transition such that dietary implications are fully understood and thus managed. On one hand, consumption of alternative proteins is driven by consumers’ increasing environmental, health and animal welfare concerns, and these alternatives may indeed offer a sustainable solution to meeting future protein needs. The potential health consequences such as nutritional deficiencies resulting from such a large-scale shift cannot be overlooked. As shown in the case of consumer calcium and iodine intake, potential nutritional deficiencies may arise from the replacement of dairy with plant-based alternatives. Moreover, these potential nutritional deficiencies may have inter-generational impacts. The research shows a critical gap in the current understanding of plant-based alternative protein diets and calls for comprehensive research to address this gap. Collaboration among researchers, policymakers, the food industry and health professionals is crucial to bridging this gap. Learnings from such collaboration can shape public health guidance to support an equitable transition to sustainable and nutritionally complete diets.

## Footnotes

1 Whilst dietary changes are reported anecdotally by consumers, there is little evidence to support large scale dietary changes.
